# Challenges in prenatal diagnosis of foetal anorectal malformation and hydrocolpos – Case report

**DOI:** 10.1016/j.amsu.2022.104949

**Published:** 2022-11-17

**Authors:** Muhammad Alamsyah Aziz, Fatima Zahra, Cut Razianti ZB, Nuniek Kharismawati, Tjut Sutjighassani, Nadia Larastri Almira, Kevin Dominique Tjandraprawira

**Affiliations:** a Department of Obstetrics and Gynecology, Faculty of Medicine, Universitas Padjadjaran – Dr. Hasan Sadikin General Hospital, Bandung, Indonesia

**Keywords:** Prenatal ultrasound, Anorectal malformation, Hydrocolpos, Ovarian cyst, Case report

## Abstract

**Introduction:**

and importance: Foetal hydrocolpos and anorectal malformation are difficult to diagnose prenatally due to abundance of differential diagnoses. This case report presents the challenges of diagnosing such disorders.

**Case presentation:**

A G3P2A0 woman came at 32 weeks of pregnancy with a referral for foetal ovarian cyst. Ultrasound revealed a singleton breech pregnancy, estimated foetal weight 3528 g. A septate abdominal cyst measuring 11.31 × 7.17 cm and polyhydramnios were present. Elective caesarean section delivered a female baby weighing 2820 g and measuring 43 cm. Neonatal examination revealed a right lateral suprapubic mass and a rectovestibular fistula. A sinoscopy revealed a suspected hydrocolpos. An abdominal hydrocolpos drainage was performed; a patent urachus and normal bilateral adnexa were present.

**Clinical discussion:**

Hydrocolpos is a rare congenital disorder due to distal obstruction of various etiologies. It may be mistaken with other pathologies, including fetal ovarian cysts. A genitourinary congenital abnormality may occur in conjunction with other abnormalities, including gastrointestinal tract anomalies. The presence of imperforate anus and/or fistula should alert the clinician of a possible association with VACTERL syndrome.

**Conclusion:**

Hydrocolpos is a rare congenital genitourinary disorder with various differential diagnoses. Simultaneous presence of other abnormalities is likely, with possible association to other syndromes.

## Highlights

• Hydrometrocolpos is a rare congenital disorder due to distal obstruction of various etiologies.
• Genitourinary congenital abnormality may occur in conjunction with other abnormalities, including gastrointestinal tract anomalies.
• The presence of imperforate anus and/or fistula may be associated with VACTERL syndrome.

## 1 Introduction

Foetal ovarian cysts (FOC) are the most common abdominal anomalies diagnosed in female fetuses with an estimated incidence of 1 in 2600 pregnancies. After 30 weeks of gestation, the most likely female abdominal anomaly is an ovarian cyst (with a high regression rate), but splenic cyst, gastrointestinal duplication, anorectal malformation, hydrometrocolpos, and liver cysts should be included in the differential diagnosis. This case report presents and discusses misdiagnosis of anorectal malformation and hydrocolpos as ovarian cyst.

## 2 Case presentation

This case is reported in line with the SCARE 2020 Guideline [1]. This case is registered at Research Registry with the following unique identifying number: researchregistry8219 (URL: https://www.researchregistry.com/register-now#home/registrationdetails/63007e3216a0ef0021cf2325/).

**Fig. 1:**
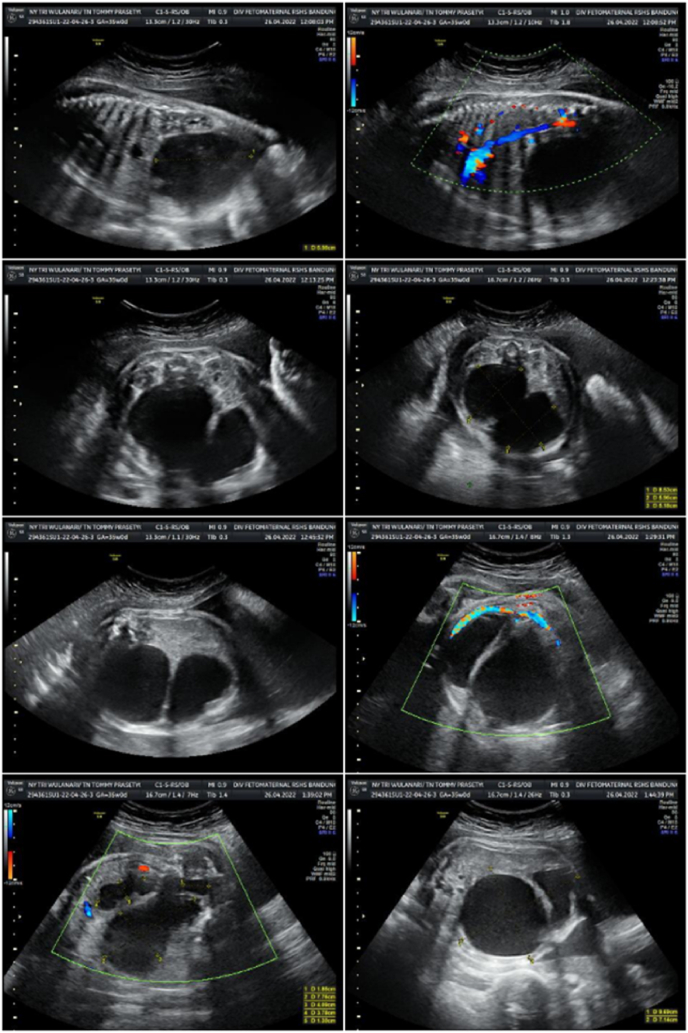
The prenatal ultrasound images at 32 weeks of gestation.

A G3P2A0 woman came to the Maternal-Foetal Medicine Unit for prenatal screening at 32 weeks of gestation. Patient came with a referral diagnosis of a foetal ovarian cyst. An obstetric ultrasound was performed, with the result of singleton pregnancy, breech presentation with estimated fetal weight (EFW) of 3528 g. On foetal abdominal ultrasound examination (Fig. 1), the stomach and both kidneys were visualized normally. Bladder was difficult to assess and an abdominal cystic mass was found with size of 11.31 × 7.17 cm with septa. The single deepest pocket (SDP) was 10.31 cm, and polyhydramnios were established. Caesarean section was performed due to breech presentation, and a female neonate was born with a body weight of 2820 g and body length of 43 cm.

**Fig. 2:**
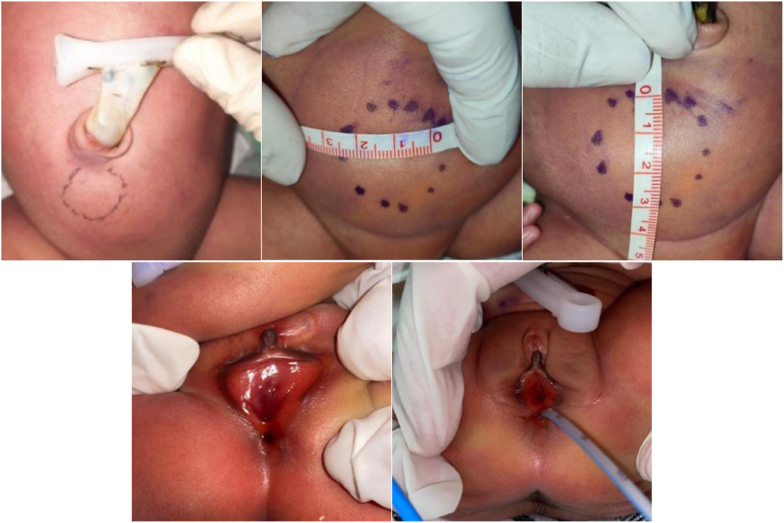
Neonatal abdominal and genital examination.

On the neonate's physical examination, a mass was found on the right lateral suprapubic area. On anogenital examination, a rectovestibular fistula was found (Fig. 2).

**Fig. 3:**
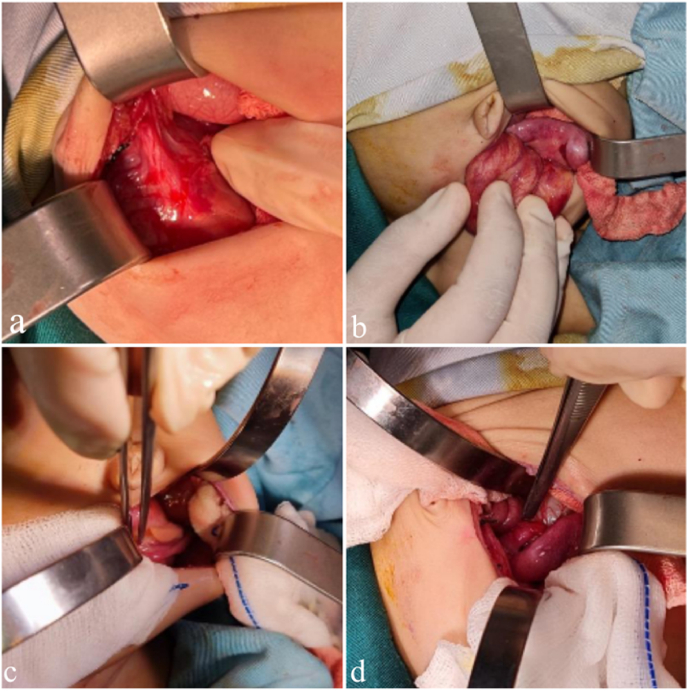
a. patent urachus; b. enlarged uterus; c. and d. bilateral Fallopian tubes within normal limits).

**Fig. 4:**
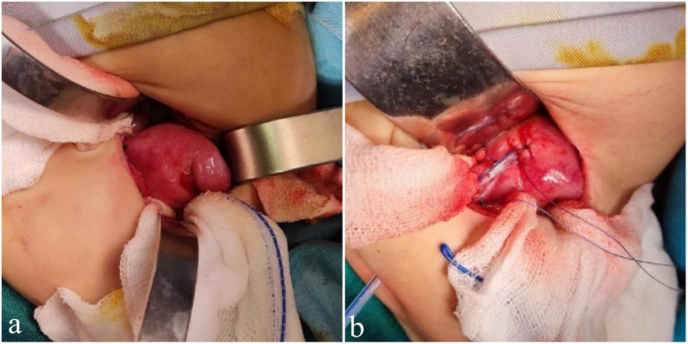
Intraoperative Findings (a. uterus with hydrocolpos; b. abdominal drainage of hydrocolpos.

After physical examination, an abdominal ultrasound was performed and a pelvic cystic mass was found with debris and internal echo was found that connected to the vagina and uterus. Liver, gallbladder and spleen ultrasound within normal limits. The baby underwent a sinoscopy with intraoperative findings of suspected hydrocolpos. Since cervical hydrocolpos drainage could not be performed, an abdominal hydrocolpos drainage was performed. Intraabdominal findings were patent urachus, with bilateral adnexa within normal limits (Fig. 3, Fig. 4).

## 3 Discussion

Foetal ovarian cysts are the most common congenital abdominal anomalies with an estimated incidence of about 1 in 2600 pregnancies. After 30 weeks of gestation, the most likely diagnosis in females is an ovarian cyst (with a high regression rate). Antenatal diagnosis of FOCs has been increased by the widespread use of ultrasound screening, with the diagnosis usually being made at around 31–32 weeks' gestation. They are usually asymptomatic and rarely accompanied with malformations, but large cysts may present with complications. Spontaneous regression of FOCs has been reported [2].

Although the pathophysiology of ovarian cysts has not yet been fully elucidated, they are usually benign functional anomalies resulting from excessive stimulation of fetal ovaries by placental and maternal hormones. Ovarian cysts are common in pregnancies complicated by maternal diabetes, pre-eclampsia or rhesus isoimmunisation. They are frequently diagnosed during the third trimester, especially after 28 weeks of gestation [2]. The following criteria are used to identify foetal ovarian cysts during foetal ultrasound examination:

1. The presence of a cystic structure, usually located in one side of foetal abdomen
2. Identification of normal genitourinary tract (kidneys, ureter and bladder)
3. Identification of a normal gastrointestinal tract (stomach, small and large bowel)
4. Female fetus

Differential diagnoses are very important and can be factored out by identifying the organ of origin, observing the anatomy and placement of the cyst. Gastrointestinal, renal, and genital anomalies are commonly misdiagnosed as ovarian cysts [3]. When an intra-abdominal cyst is found in a female fetus, the possible differential diagnoses are renal cysts (unilocular or multiple) that are found in the renal area, with or without alterations of the kidneys architecture; hydronephrosis and/or ureterocele which are often associated with the first being described as a mass adjacent to the vertebral column distorting the renal pelvis and the latter located by entry of the bladder. In addition, hydrocolpos, described as a mass posterior to the bladder often associated with fetal uterine dilation, must be ruled out.

Other differential diagnosis that are commonly associated with FOCs are urachal cyst, intestinal duplication abnormalities, hydrometrocolpos, liver cysts and mesenteric cysts [3]. An intestinal duplication cyst appears as a hypoechoic muscular layer surrounding a hyperechoic mucosal layer and tends to be more tubular or ovoid in shape than other lesions. A urachal cyst is generally located in the midline and extends to the umbilicus. A large and overdistended bladder or dilated intestinal loops can also mimic FOCs. Mesenteric cysts usually occur in the mid-abdominal region and are characteristically observed to be mobile on ultrasound examination. In gastrointestinal abnormalities, concomitant foetal ascites or polyhydramnios may also be present [3]. Diagnosis of a foetal ovarian cyst is usually presumptive because mesenteric cysts, or enteric duplications cannot be ruled out with absolute certainty prenatally. Factors more indicative of a foetal ovarian cyst include bilaterality, echogenicity, and cyst septations [4].

In this case, the patient was previously referred with the diagnosis of foetal ovarian cysts. Only after the physical examination was done with the findings of rectovestibular fistula, that another differential diagnosis other than FOC was finally evaluated. An abdominal ultrasound revealed a pelvic cystic mass with debris and internal echo that connected to the vagina and uterus. The neonate then underwent a sinoscopy with intraoperative findings of suspected hydrocolpos. Hemato/hydrocolpos is a condition in which menstrual blood or secretory fluid accumulates in the vagina due to vaginal obstruction caused by congenital urogenital anomalies or acquired vaginal occlusion due to infection, trauma, or sexual abuse. Hydrocolpos due to congenital urogenital anomalies are a rare condition which make diagnosis is often missed or delayed due to its rare incidence and nonspecific symptoms. Hydrometrocolpos occurs in the female fetus due to dilatation of the uterus and vagina as a result of mucus secretions in the genital tract. When only the vagina is distended, it is termed as hydrocolpos, but if there is associated uterine distension it is termed as hydrometrocolpos. After abdominal hydrocolpos drainage was performed in this case and an enlarged uterus with hydrocolpos was found, the diagnosis shifted into hydrometrocolpos.

There is no data on fetal presentation of hydrometrocolpos due to the rarity of the disease and the difficulty in prenatal diagnosis. The prevalence of congenital dilatation of the uterus and vagina causing hydrometrocolpos is less than 1 per 30,000 births with the first report published in 1856 [5,6].

Hydrocolpos may be caused by an imperforate hymen, a transverse vaginal membrane, vaginal atresia or cloacal malformation. In case of a cloacal malformation, there is a common channel for the urethra, vagina and anus. The obstruction of urinary, genital and intestinal secretions caused by these malformations blocks drainage, leading to dilatation of the proximal structures. The content of fluid accumulating may be clear (urine) or turbid with a fluid-debris level on sonography (meconium or cervical secretions. The spectrum of hydrocolpos is quite broad, ranging from mild cases that may remain undetected until adolescence to more severe conditions described prenatally as a large pelviabdominal cystic mass, just like in the case [6].

Most cases of hydrometrocolpos are sporadic, but the condition may occur following prenatal dexamethasone treatment for congenital adrenal hyperplasia. It has also been associated with ambiguous genitalia in 45,X/46,XY mosaicism, and Mckusick–Kaufman syndrome, which involves multiple malformations and is characterized by hydrometrocolpos and polydactyly. In such cases the hydrometrocolpos is due to either cervical or vaginal atresia and not to a persistent urogenital sinus. The presence of a persistent urogenital sinus has been found to be associated with uterine anomalies (bicornuate uterus, cervical atresia), vaginal atresia or duplication, hydronephrosis, renal agenesis, imperforate anus, polycystic kidneys, esophageal atresia and sacral hypoplasia [7].

Prenatally the presence of a fluid–debris level inside a pelvic cystic anechoic mass must be considered a crucial finding. Multiple internal echoes are due to thick mucoid cervical or vaginal secretions. This finding must alert the treating clinician to the possibility of the presence of a persistent urogenital sinus. Prenatal cystic dilatation of the vagina is often misdiagnosed as a distended bladder, as the bladder is displaced anteriorly by the vagina making it difficult to identify. Prenatal ultrasound therefore has a definitive role in detecting an obstructed genital tract, allowing rapid postnatal treatment to drain the vagina and relieve the urinary tract obstruction [7]. Hydrocolpos commonly associated with a vast spectrum of urorectal septum malformations. Therefore a throughout sonographic examination including identification of a normal anal canal and rectum are important for prenatal counselling.

If early diagnosis and treatment are done early, the long-term outcomes generally yield a good result. But, without early and proper treatment, late complications such as tubal infection, adhesion, pelvic endometriosis, infertility, and renal failure secondary to hydronephrosis can develop. Ultrasonography, computed tomography (CT) and magnetic resonance imaging (MRI) used to evaluate patients with hydrocolpos. MRI is preferred because of its high-resolution, soft tissue contrast, and provides useful information for the differential diagnosis of obstructive causes and management decisions. Surgical treatment is necessary for patients with hydrocolpos due to congenital vaginal obstruction. Although drainage of accumulated blood or secretions in the vagina should be done, long-term effect of transvaginal drainage must be considered because it may increase the risk of retrograde infection [6].

When physical examination of this case was performed, a mass was found on the right lateral suprapubic area. On anogenital examination, a rectovestibular fistula with an imperforate anus was found. Anomalies of the anus and rectum have usually been explained as caused by the caudal descent of the urorectal septum toward the cloacal membrane between 4 and 8 weeks of gestation. At 4–6 weeks the cloacal membrane becomes partitioned into the anterior urogenital sinus and the posterior anorectum by the cranial to caudal growth of mesoderm-derived urorectal septum. The mesoderm-derived urorectal septum is composed of the midline Tournex fold and two lateral Rathke folds. The lower third of the anal canal is derived from the ectoderm of the anal pit. Failure of Rathke folds to develop results in arrest of the inferior urorectal septum, resulting in a persistent cloaca in the female. This arrest in Rathke folds usually occurs just below the paramesonephric duct, but a more caudal arrest in Rathke folds could result in a high rectovaginal fistula in a female. In the female it is more likely to result in a cloacal anomaly or duplicated vagina and uterus. The malalignment of Tourneaux and Rathke folds may also result in vestibular fistula in females. Despite normal descent of Tourneaux and Rathke folds, the lack of an ectodermal anal pit results in imperforate anus. Failure of the anal membrane to resorb or incomplete resorption despite formation of the anal pit results in rectal atresia or stenosis, respectively [4].

Imperforate anus is a common congenital anomaly with an incidence of 1:1,500 to 1:5,000 in neonates. It may either be a solitary anomaly without any malformation or, in many cases, be associated with multiple congenital anomaly subsets, such as VACTERL association or trisomy 21. Imperforate anus, with other serious anomalies, can lead to significant morbidity and mortality [8].

Although imperforate anus is a relatively common malformation, it cannot be accurately diagnosed prenatally. Imperforate anus can occur as a solitary abnormality but is likely to have accompanying malformations. Hence, when a foetal imperforate anus is suspected, referral to a tertiary center that can provide accurate diagnosis and adequate management is necessary. Several factors, including the presence of an anocutaneous or anovestibular fistula and well developed anal sphincters, which can be visualized as an image similar to the normal anal sphincter and canal mucosa, may account for difficulties in diagnosing certain cases of imperforate anus. Conversely, even if one malformation associated with vertebral defects, anal atresia, cardiac defects, tracheo-esophageal fistula, renal anomalies, and limb abnormalities (VACTERL) association is detected prenatally, more detailed ultrasonography examinations should be performed regarding the presence of imperforate anus.

There are three types of imperforate anus according to the distal rectal pouch and the puborectalis muscle [1]: the high-type, in which the distal pouch ends above the puborectalis muscle [2]; the intermediate-type, in which the pouch ends at the puborectalis muscle; and [3] the low-type, in which the pouch ends through the puborectalis muscle. Traditionally, imperforate anus is diagnosed by prenatal ultrasound on detecting the presence of a dilated distal bowel, or rectum or intraluminal meconium calcification, or enterolithiasis; however, it is not always suspected with the presence of a fistula [9]. A recent study suggested that a low-type imperforate anus is suspected if the size of the anus is small or the distance between the anus and the genitalia is short. The high-type imperforate anus, however, is relatively well diagnosed during pregnancy and is more frequently found in male infants, with a higher mortality and morbidity compared to other types. In contrast, low-type is more common in female fetuses, with a relatively good prognosis; however, it is difficult to recognize in the prenatal period [9].

Even without considering other accompanying anomalies (except imperforate anus), the exact anatomical type of atresia and the existence of a fistula should be carefully examined. This is important because it determines the appropriate timing for corrective surgery as well as the surgical stages. Urgent reconstructive anorectal surgery is not necessary; however, immediate evaluation is important, and urgent decompressive surgery may be necessary. If a diagnosis is suspected prenatally, it is essential for the surgeon to provide appropriate guidance to the pregnant woman and to make a delivery plan to prepare for the possibility of neonatal operation. Thus, detecting the presence of an imperforate anus via prenatal ultrasonography is important for obstetricians, pediatric surgeons, and pregnant women to plan for an early treatment [10].

## 4 Conclusion

Hydrometrocolpos is a rare congenital disorder due to distal obstruction of various etiologies. It may be mistaken with other pathologies, including fetal ovarian cysts. A genitourinary congenital abnormality may occur in conjunction with other abnormalities, including gastrointestinal tract anomalies. The presence of imperforate anus and/or fistula should alert the clinician of a possible association with VACTERL syndrome.

## Annals of medicine and surgery

The following information is required for submission. Please note that failure to respond to these questions/statements will mean your submission will be returned. If you have nothing to declare in any of these categories then this should be stated.

## Ethical approval

The institutional review board has determined that our study is exempt from ethical approval as it is a review.

## Sources of funding

The authors declared that we did not receive any external funding for our study.

## Author contribution

All authors were responsible for the conception and design of the study. FZ, CRZB, and NK collected patient data. MAA, TS, NLA, and KDT drafted the manuscript. All authors reviewed and approved this final version for publication.

## Registration of research studies

The study's registration on researchregistry.com is researchregistry8219.

(https://www.researchregistry.com/register-now#home/registrationdetails/63007e3216a0ef0021cf2325/).

## Guarantor

Muhammad Alamsyah Aziz as the first author is the guarantor of this study.

## Consent

Written informed consent was obtained from the patient for publication of this case report and accompanying images. A copy of the written consent is available for review by the Editor-in-Chief of this journal on request.

## Provenance and Peer Review

Not commissioned, externally peer-reviewed.

## Declaration of competing interest

The authors declare that we do not have any conflicts of interests.
